# The Association of Physical Activity With Overweight/Obesity and Type 2 Diabetes in Nepalese Adults: Evidence From a Nationwide Non‐Communicable Disease Risk Factor Survey

**DOI:** 10.1002/osp4.70046

**Published:** 2025-01-19

**Authors:** Ritesh Chimoriya, Kritika Rana, Jonas Adhikari, Sarah J. Aitken, Prakash Poudel, Aayush Baral, Lal Rawal, Milan K. Piya

**Affiliations:** ^1^ School of Medicine Western Sydney University Campbelltown Australia; ^2^ Philanthropy Research Collaboration Auburn Australia; ^3^ Concord Institute of Academic Surgery Concord Repatriation General Hospital Concord Australia; ^4^ Faculty of Health and Medicine The University of Sydney Sydney Australia; ^5^ Translational Health Research Institute Western Sydney University Penrith Australia; ^6^ School of Health Sciences Western Sydney University Penrith Australia; ^7^ Office of Research and Education Canberra Health Services ACT Government Garran Australia; ^8^ Department of Public Health Torrens University Australia Melbourne Australia; ^9^ School of Health, Medical and Applied Sciences Central Queensland University Sydney Australia; ^10^ Physical Activity Research Group Appleton Institute Central Queensland University Norman Gardens Australia; ^11^ Macarthur Diabetes Endocrinology and Metabolism Service Camden and Campbelltown Hospitals Campbelltown Australia

**Keywords:** diabetes, non‐communicable disease, obesity, overweight, physical activity, risk factor, type 2 diabetes

## Abstract

**Background:**

The rising prevalence of obesity and type 2 diabetes (T2DM) is a significant public health concern, particularly in low‐ and middle‐income countries. This study aimed to explore the association between physical activity levels, overweight/obesity, and T2DM in a nationwide survey of Nepalese adults.

**Methods:**

This was a secondary analysis of the 2019 non‐communicable diseases (NCD) risk factors STEPS survey conducted in Nepal. Demographic and anthropometric data, body mass index (BMI) and T2DM status were collected along with assessment of physical activity using Global Physical Activity Questionnaire (GPAQ). A two‐stage data analysis was conducted, first using descriptive statistics to summarize participant characteristics and differences across BMI and T2DM status, and then applying multivariate analyses to assess associations between physical activity levels, BMI and T2DM.

**Results:**

Of the 5284 participants included, 28.0% had overweight/obesity, 5.8% had obesity, and 6.5% had T2DM. The mean age of the participants was 40.1 years (95% CI: 39.8–40.6), and 63.9% were female. The overall physical activity energy expenditure was higher in the lean group (BMI < 25 kg/m^2^) compared to the those with overweight/obesity, and among participants without T2DM compared to those with T2DM. Sedentary behavior was more common among individuals with overweight/obesity and T2DM. A higher proportion of participants with low physical activity was observed in the overweight/obesity group compared to the lean group (8.9% vs. 6.3%) and the T2DM group compared to the non‐T2DM group (11.7% vs. 6.7%). Low physical activity was associated with overweight/obesity (OR:1.4; 95% CI:1.1–1.8), obesity (OR:2.1; 95% CI:1.5–2.3), T2DM (OR:1.6; 95% CI:1.1–2.3) and T2DM in the presence of obesity (OR:3.6; 95% CI:1.7–7.8).

**Conclusion:**

This study highlights the low rates of physical activity and higher rates of sedentary behavior among adults with overweight/obesity and T2DM in Nepal. Public health interventions promoting physical activity and reducing sedentary behavior may help reduce the burden of these NCDs.

## 1 Introduction

The rapidly increasing prevalence of overweight, obesity, and type 2 diabetes (T2DM) presents a global public health challenge [1, 2]. Current estimates suggest that nearly one‐third of all adults fall short of engaging in adequate physical activity, a contributory factor in the pathogenesis of obesity and associated non‐communicable diseases (NCDs), including T2DM [3]. The International Diabetes Federation (IDF) reports that approximately 537 million adults aged 20–79 years are living with diabetes, with approximately 90% having T2DM, and projections indicate an increase to 643 million by 2030 and 783 million by 2045 [4].

The causes of this increasing global prevalence of overweight/obesity are multifactorial, with reduced physical activity and increased consumption of energy dense foods being contributory factors [5, 6, 7]. This has been attributed to sedentary lifestyles, rapid urbanization, and evolving transportation methods [8, 9, 10]. Obesity‐induced insulin resistance is further exacerbated by nonesterified fatty acids (NEFAs), glycerol, cytokines, and proinflammatory mediators secreted from adipose tissues [11, 12, 13]. The interplay of insulin resistance and impaired beta cell function culminates in T2DM [12]. Physical activity is a modifiable factor that can reverse obesity‐induced insulin resistance and help prevent T2DM [14]. Moreover, physical activity or exercise has been shown to reduce muscle insulin resistance independent of weight, while also contributing to cardiovascular risk reduction [15, 16]. For individuals with T2DM, physical activity is often recommended to prevent obesity or to achieve weight loss in those with both obesity and T2DM [16]. Integrating adequate physical activity with dietary modifications, leading to a 5%–7% reduction in body weight, may further assist in maintaining glucose levels in individuals with T2DM [17, 18]. Furthermore, sedentary behavior has been associated with increased risks of cardiovascular disease, T2DM, and all‐cause mortality [19].

Although much research has focused on high‐income countries, low‐ and middle‐income countries (LMICs) like Nepal face unique challenges that amplify the impact of overweight/obesity and T2DM on public health. In Nepal, the burden of NCDs such as T2DM has significantly increased due to epidemiological shifts marked by unplanned urbanization, increasing sedentary lifestyles, rising obesity rates, and inadequate physical activity that present significant challenges to the national healthcare system [7, 20]. While screening for diabetes is not routinely available, the reported prevalence of diabetes among Nepalese adults aged 20–79 years was 4% in 2017 and is projected to increase to 6.1% by 2045 [20]. A parallel trend is observed with the prevalence of overweight/obesity rising steadily from 21.7% in 2013 to 24.3% in 2019 [7, 21]. Despite evidence suggesting the benefits of regular physical activity in reducing complications and mortality associated with NCDs, South Asian countries, including Nepal, frequently exhibit low levels of physical activity [22, 23]. Moreover, the prevalence of insufficient physical activity among Nepalese adults is on an increasing trend [22, 24]. Understanding these trends is important to help inform targeted interventions to promote physical activity in resource‐constrained settings, especially for LMICs such as Nepal, where limited healthcare resources and infrastructure further compound the public health impact of NCDs linked to overweight/obesity as well as T2DM. However, to the best of our knowledge, there are no comprehensive population‐based studies examining the interrelationship between physical activity, overweight/obesity and T2DM in Nepal. We hypothesized that low physical activity and increased sedentary behavior would be associated with higher rates of overweight/obesity and T2DM among adults in Nepal. Therefore, this study aimed to investigate the relationship between physical activity, overweight/obesity, and T2DM among Nepalese adults using a nationally representative sample from the World Health Organization (WHO) STEPwise approach to NCD risk‐factor surveillance (STEPS) survey in Nepal.

## 2 Methods

### 2.1 Study Design and Data Sources

This study was a secondary analysis of the nationally representative cross‐sectional NCD risk factors survey conducted between February 2019 and May 2019, following the World Health Organization (WHO) STEPwise approach to surveillance (STEPS), an integrated surveillance tool used to collect, analyze and disseminate core standardized information on NCDs [21, 25]. The survey utilized a multistage cluster sampling method to select 6475 eligible participants aged 15–69 years across all seven provinces (geographical regions) in Nepal. The primary sampling units (PSUs) were 259 wards (geo‐political municipal subdivision), with 37 PSUs from each province, selected using a probability proportional to size (PPS) method. Systematic random sampling was employed to select 25 households per PSU, and one adult member aged 15–69 years from each household was randomly sampled to participate in the survey. The data sources and methodology have previously been described in detail [25, 26, 27].

### 2.2 Data Collection Measures

An interviewer‐administered questionnaire technique was used to conduct face‐to‐face interviews with the participants, collecting information on their sociodemographic and lifestyle characteristics. The survey was carried out in accordance with the STEPS protocol, employing recommended instruments and conducted in different steps to collect information related to behavioral factors (STEP I), physical measurements (STEP II) and biochemical measures (STEP III) [26, 27, 28]. Data were collected electronically through Android devices using the STEPS application developed by the WHO STEPS team [28].

#### 2.2.1 Physical Activity

Physical activity was assessed in STEP I using the previously validated Global Physical Activity Questionnaire (GPAQ) Version 2 [29, 30]. The GPAQ was used to collect information on respondents' participation in different physical activities across three domains [31]. It included the work‐related physical activity domain, which assesses the frequency and duration of vigorous and moderate activities conducted as part of occupational duties, such as lifting or carrying heavy loads. The transport‐related physical activity domain focuses on physical movements associated with commuting, including walking and cycling, and considers the regularity and length of these activities. Leisure‐time physical activity is the third domain, evaluating the frequency and duration of physical activities such as sports and fitness exercises during free time, ranging from moderate to vigorous intensity. Additionally, the GPAQ assesses sedentary behavior by collecting data about the amount of time spent sitting or reclining during typical daily activities such as desk work or watching TV.

The data collected in the survey includes the duration (in minutes) and frequency (days) of walking, moderate‐intensity, and vigorous‐intensity activities performed for at least 10 min per session during the previous 7 days across the domains. In accordance with the GPAQ analysis guide, the responses were converted to metabolic equivalent of task and expressed in MET‐minutes/week [31, 32]. Metabolic equivalents (METs) are physiological measures expressing the energy cost of physical activities, defined as the ratio of metabolic rate (and therefore the rate of energy consumption) during a specific physical activity to a reference metabolic rate. The METs min/week of a specific activity (vigorous, moderate, and low‐intensity physical activity [PA]) was calculated by multiplying the MET value of a particular activity (4.0 for walking and moderate PA, and 8.0 for vigorous PA) by the minutes spent doing that activity. Physical activity was then calculated using the formula = MET × activity days per week (d) × daily activity time (min). For example, walking METs min/week = 4 × walking minutes × walking days. According to the total physical activity and the frequency of different types of physical activities within 1‐week, physical activity was categorized into high, moderate, and low, as shown in Table 1. To classify individuals into high, moderate, or low physical activity categories, meeting any one of the criteria for each level was sufficient.

**TABLE 1:** Classification of physical activity levels.

Physical activity	Description
High physical activity	• Vigorous‐intensity activity for at least 3 days, achieving a minimum total physical activity of at least 1500 MET‐minutes/week.
	• 5 or more days of any combination of walking, moderate‐intensity, or vigorous‐intensity activities, achieving a minimum total physical activity of at least 3000 MET‐minutes/week.
Moderate physical activity	• At least 20 min of vigorous‐intensity activity per day for 3 or more days per week.
	• At least 30 min of moderate‐intensity activity per day for 5 or more days per week.
	• 5 or more days of any combination of walking, moderate‐intensity, or vigorous‐intensity activities, achieving a minimum total physical activity of at least 600 MET minutes/week.
Low physical activity	• Insufficiently active (some physical activity but below the criteria for high or moderate level).
	• Inactive (no reported physical activity).

#### 2.2.2 Physical Measurements

Physical measurements were undertaken in STEP II, which included height (in cm) using a portable standard stature tape, weight (in kg) using a SECA weighing machine, waist and hip circumference (in cm) using a constant tension body tape, and blood pressure (in mmHg) using an OMRON blood pressure monitor. Body Mass Index (BMI) was calculated as weight in kilograms (kg) divided by the square of height in meters (kg/m^2^). BMI was categorized as: underweight (< 18.5 kg/m^2^), normal weight (18.5 to < 25 kg/m^2^), overweight (25 to < 30 kg/m^2^), and obesity (≥ 30 kg/m^2^). A BMI of ≥ 25 kg/m^2^ was used as the criterion to determine the prevalence of overweight/obesity.

#### 2.2.3 Biochemical Measures

After completing STEP I and STEP II of data collection at sampled individuals' homes, biochemical assessments were conducted the following day at a designated location for the PSU in STEP III. Blood samples for measuring blood glucose and total cholesterol were collected using CardioCheck PA point‐of‐care testing (POCT) equipment (dry chemistry) [21]. Concentrations of glucose and total cholesterol were measured in capillary whole blood. Fasting samples were taken to measure raised blood glucose, and participants were instructed to fast overnight for 12 h at the time of the household visit. As per the criteria included in the national STEPS survey report, participants with a blood glucose level ≥ 126 mg/dL (7.0 mmol/L) or currently taking medication for raised blood glucose were considered to have T2DM [21]. Participants with a total cholesterol ≥ 190 mg/dL (5.0 mmol/L) or currently taking medication for increased cholesterol were considered to have raised cholesterol [21].

### 2.3 Data Analysis

A descriptive analysis was conducted to describe the overall distribution of sociodemographic characteristics, physical measurements, and biochemical profiles of the participants across the groups by BMI and presence of T2DM. This involved calculating frequencies, proportions, means, and standard deviations (SD) for each parameter. Domain‐specific physical activity (work, transport, and leisure‐time), total MET‐minutes per week and sedentary behavior were assessed using median and interquartile ranges (IQR) in relation to BMI groups and T2DM status. An independent sample median test was applied to determine the statistical significance of differences in GPAQ domains observed between groups. The association between low physical activity and the presence of overweight/obesity and T2DM was analyzed using multivariate logistic regression. Age and gender were adjusted for in the multivariate logistic regression model to control for their potential confounding effects on the relationship between physical activity and BMI or T2DM status. The magnitude of these associations was measured using the adjusted odds ratio (AOR) and 95% confidence intervals (CI), with a *p*‐value of < 0.05 considered statistically significant. Data quality checks, including handling of missing data and validation of GPAQ responses, were conducted prior to analysis. Missing values were addressed using pairwise deletion. Data analysis was carried out in two stages using the Statistical Package for the Social Sciences, Version 29 (SPSS for MacOS, SPSS Inc., Chicago, IL, USA).

### 2.4 Ethics Approval

The NCD risk factors STEPS survey, from which the data for this analysis were extracted, received ethics approval from the Ethical Review Board of the Nepal Health Research Council (approval number: 293/2018). License for the secondary data analysis for this study was approved on 03/02/2022 (approval ID: 1478).

## 3 Results

### 3.1 Sociodemographic Characteristics of Participants

Of the 6475 eligible participants invited to participate, 5593 adults aged 15–69 years agreed to participate, resulting in a response rate of 86.4%. Anthropometric data were not available for 74 participants, and biochemical measures for T2DM history were not available for 235 participants, resulting in the inclusion of 5284 participants in the final analysis. The mean age of the participants was 40.1 years (95% CI: 39.8–40.6) and 63.9% (*n* = 3377) were female. The detailed characteristics of the study participants are presented in Table 2. Of the total sample (*n* = 5284), 72.0% had a BMI < 25 kg/m^2^ (lean group), 28.0% (*n* = 1478) had overweight/obesity (BMI ≥ 25 kg/m^2^), 5.8% (*n* = 304) had obesity (BMI ≥ 30 kg/m^2^), and 6.5% (*n* = 342) had T2DM. The prevalence of low physical activity in the sample was 7.0% (*n* = 371).

**TABLE 2:** Sociodemographic characteristics, physical measurements, and biochemical profiles of the study participants.

Characteristics	All participants (*n* = 5284)	Moderate or high physical activity (*n* = 4913)	Low physical activity (*n* = 371)
Sociodemographic characteristics (*n* (%))			
*Age group*			
15–29 years	1337 (25.3%)	1265 (25.7%)	72 (19.4%)
30–44 years	1930 (36.5%)	1814 (36.9%)	116 (31.3%)
45–69 years	2017 (38.2%)	1834 (37.3%)	183 (49.3%)
*Age (continuous; years)*	40.2 ± 14.0	39.9 ± 14.0	44.2 ± 15.0
*Gender*			
Male	1907 (36.1%)	3156 (64.2%)	221 (59.6%)
Female	3377 (63.9%)	1757 (35.8%)	150 (40.4%)
*Education*			
No formal or less than primary schooling	590 (11.2%)	558 (18.6%)	32 (14.6%)
Primary school completed	993 (18.8%)	924 (30.7%)	69 (31.7%)
Secondary school completed	1025 (19.4%)	952 (31.7%)	73 (33.5%)
High school completed	451 (8.5%)	424 (14.1%)	27 (12.4%)
Higher education completed	164 (3.1%)	148 (4.9%)	17 (7.8%)
*Marital status*			
Never married	505 (9.6%)	480 (9.8%)	25 (6.7%)
Currently married	4490 (85.0%)	4171 (84.9%)	319 (86.0%)
Divorced/Widowed/Separated	288 (5.5%)	262 (5.3%)	27 (7.2%)
*Currently in paid employment*			
Yes	1613 (30.5%)	1475 (30.1%)	138 (37.2%)
No	3665 (69.4%)	3438 (69.9%)	233 (62.8%)
*Province (Geographic region)*			
Koshi	757 (14.3%)	721 (14.7%)	36 (9.7%)
Madhesh	768 (15.5%)	697 (14.2%)	71 (19.1%)
Bagmati	714 (13.5%)	652 (13.3%)	62 (16.7%)
Gandaki	760 (14.4%)	698 (14.2%)	62 (16.7%)
Lumbini	756 (14.3%)	713 (14.5%)	43 (11.6%)
Karnali	754 (14.3%)	729 (14.8%)	25 (6.7%)
Sudurpaschim	775 (14.7%)	703 (14.3%)	72 (19.4%)
Physical measurements and biochemical profile (mean ± SD)			
Weight (kg)	55.5 ± 11.4	55.3 ± 11.2	58.0 ± 13.8
Body mass index (BMI) (kg/m^2^)	23.1 ± 4.4	23.1 ± 4.3	24.0 ± 5.4
Waist to hip ratio (WHR)	0.9 ± 0.1	0.9 ± 0.1	0.9 ± 0.1
Systolic blood pressure (mmHg)	126.6 ± 17.9	128.3 ± 18.7	131.3 ± 23.7
Diastolic blood pressure (mmHg)	83.4 ± 10.9	84.4 ± 11.6	85.4 ± 13.9
Fasting blood glucose (mg/dL)	93.9 ± 27.7	93.4 ± 27.0	100.9 ± 35.1
Total cholesterol (mg/dL)	144.6 ± 38.5	144.3 ± 38.2	148.1 ± 41.3

### 3.2 Physical Activity Levels, BMI and T2DM

Table 3 presents physical activity levels and sedentary behavior in relation to BMI and T2DM status. In the work domain, vigorous MET minutes per week showed a significant difference (*p* < 0.001), with higher MET minutes in the lean group (BMI < 25) compared to the overweight/obesity group (BMI ≥ 25). Similarly, people without T2DM had higher moderate and vigorous MET minutes per week (*p* < 0.001) compared with those with T2DM. The same pattern was noted in the transport domain, where transport activity MET minutes per week showed significant differences between BMI groups (*p* < 0.001) and between participants with and without T2DM (*p* < 0.001). Sedentary minutes per day was significantly higher in the overweight/obesity group compared to the lean group (*p* = 0.002), and in people with T2DM compared to those without T2DM (*p* = 0.002). The total energy expenditure with physical activity was higher in the lean group compared to the overweight/obesity group (*p* < 0.001), and in people without T2DM compared to those with T2DM (*p* < 0.001). A higher proportion had low physical activity in the overweight/obesity group compared to the lean group (8.9% vs. 6.3%; *p* < 0.001) and in the group with T2DM compared to those without T2DM (11.7% vs. 6.7%; *p* < 0.001).

**TABLE 3:** Physical activity levels and sedentary behavior in relation to body mass index and type 2 diabetes (T2DM) status among study participants.

		All participants	Body mass index (BMI) in kg/m^2^			Type 2 diabetes (T2DM)		
			Lean group (BMI < 25 kg/m^2^)	Overweight/obesity group (BMI ≥ 25 kg/m^2^)	*p*‐value	Without T2DM	With T2DM	*p*‐value
*Domain: Work*								
Moderate MET minutes per week	Median; IQR	2080; 3480	2160; 3480	1902; 3105	0.104^a^	2160; 3480	1440; 3360	< 0.001^b^
	Mean ± SD	2911.5 ± 3038.4	2944.1 ± 3039.7	2827.7 ± 3034.5		2970.3 ± 3074.1	2063.0 ± 2307.8	
Vigorous MET minutes per week	Median; IQR	0; 6720	0; 6720	0; 3840	< 0.001^a^	0; 6720	0; 1440	< 0.001^b^
	Mean ± SD	4188.0 ± 6729.9	4605.9 ± 6995.2	3111.8 ± 5859.5		4330.7 ± 6814.1	2126.2 ± 4934.0	
*Domain: Transport*								
Transport activity MET minutes per week	Median; IQR	1680; 1960	1680; 2240	960; 1320	< 0.001^a^	1680; 1960	1080; 1120	< 0.001^b^
	Mean ± SD	1854.5 ± 1985.8	2011.3 ± 2114.1	1450.9 ± 1537.6		1881.5 ± 2012.4	1465.0 ± 1501.3	
*Domain: Leisure*								
Moderate MET minutes per week	Median; IQR	0; 0	0; 0	0; 0	—	0; 0	0; 0	—
	Mean ± SD	201.0 ± 698.9	205.8 ± 682.3	188.6 ± 740.0		203.4 ± 707.4	166.5 ± 561.4	
Vigorous MET minutes per week	Median; IQR	0; 0	0; 0	0; 0	—	0; 0	0; 0	—
	Mean ± SD	157.0 ± 956.7	188.6 ± 740.3	74.6 ± 589.0		160.9 ± 976.0	103.6 ± 611.9	
*Sedentary behavior*								
Sedentary minutes per day	Median; IQR	120; 240	120; 240	150; 210	0.002^a^	120; 240	180; 240	0.002^b^
	Mean ± SD	205.3 ± 182.9	202.1 ± 182.8	213.3 ± 183.0		203.4 ± 182.6	231.5 ± 185.6	
*Combined indicators*								
Sum of all activity per week	Median; IQR	6480; 10,260	6840; 10,800	5040; 8400	< 0.001^a^	6720; 10,440	3480; 6020	< 0.001^b^
	Mean ± SD	9312.2 ± 9120.5	9956.3 ± 9423.7	7653.6 ± 8058.0		9546.7 ± 9234.1	5924.3 ± 6395.8	
Low physical activity		371 (7.0%)	239 (6.3%)	132 (8.9%)	< 0.001^c^	331 (6.7%)	40 (11.7%)	< 0.001^d^

^a^ *p*‐value of the independent samples median test (lean group vs. overweight/obesity group).

^b^ *p*‐value of the independent samples median test (without T2DM vs. with T2DM).

^c^ *p*‐value of the chi‐square test (lean group vs. overweight/obesity group).

^d^ *p*‐value of the chi‐square test (without T2DM vs. with T2DM).

### 3.3 Low Physical Activity, Overweight/Obesity and T2DM

**FIGURE 1:**
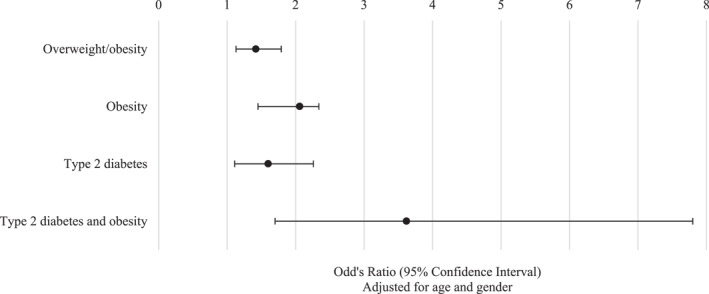
Association of low physical activity with overweight, obesity and type 2 diabetes.

As shown in Figure 1, low physical activity was significantly associated with overweight/obesity (BMI ≥ 25) (OR: 1.4; 95% CI: 1.1–1.8; *p* = 0.002), obesity (BMI ≥ 30) (OR: 2.1; 95% CI: 1.5–2.3; *p* < 0.001), T2DM (OR: 1.6; 95% CI: 1.1–2.3; *p* = 0.011) and T2DM in the presence of obesity (OR: 3.6; 95% CI: 1.7–7.8; *p* = 0.001).

## 4 Discussion

The present study provides evidence on the association between physical activity, obesity, and T2DM among the adult population of Nepal based on WHO STEPS NCD Risk‐Factor Surveillance data. This study reports a high prevalence of overweight/obesity (28.0%), obesity (5.8%), and T2DM (6.5%) among Nepalese adults. This study also demonstrates the high prevalence of low physical activity (7.0%) across the adult population, with it being significantly higher in people with overweight/obesity or with T2DM, highlighting the complex interplay between lifestyle behaviors and metabolic health in shaping the burden of NCDs [33]. The odds of having T2DM in the presence of obesity were significantly higher among individuals with low physical activity, underscoring the multiple impact of these risk factors [34]. These findings align with global trends and may be explained by the fact that insufficient physical activity not only predisposes individuals to overweight/obesity but also to insulin resistance and the risk of T2DM [35, 36]. The term “diabesity” aptly describes the dual burden of obesity and T2DM observed in this study, with a higher prevalence of low physical activity noted among individuals with overweight/obesity and T2DM compared to the lean group or those without T2DM [37, 38].

Participants in the lean group (BMI < 25) demonstrated higher physical activity levels compared with those in the overweight/obesity group (BMI ≥ 25), which may indicate a potential effect of regular physical activity against overweight/obesity. This finding aligns with the widely recognized role of physical activity in energy expenditure, weight management, and metabolic health [33, 34]. Similarly, participants without T2DM demonstrated higher physical activity levels compared with those with T2DM, which may suggest a potential link with existing evidence that identifies physical inactivity as a modifiable risk factor for NCDs [33, 34]. A higher proportion of individuals with low physical activity was observed in the overweight/obesity group (8.9% vs. 6.3%) and T2DM group (11.7% vs. 6.7%) compared to their respective counterparts. The high prevalence of sedentary behavior among individuals with overweight/obesity and those with T2DM suggests a possible contributing factor to the observed rates of obesity and T2DM in this population. Sedentary behavior has been implicated in the pathogenesis of obesity and T2DM, and our findings emphasize the need to address and reduce sedentary time as part of comprehensive preventive strategies [7, 19, 39]. Overall, these findings highlight the importance of promoting regular physical activity as a pivotal component of public health interventions aiming to mitigate the dual burden of overweight/obesity and T2DM [34, 40].

This study revealed a higher level of self‐reported physical activity in the work domain compared with transport and leisure domains, possibly reflecting the labor‐intensive nature of many jobs in Nepal. Previous studies suggest that individuals who are highly active at work often engage less in physical activity during leisure time [41, 42], which could be a plausible explanation for the low levels of leisure‐time physical activity observed in this study. Additionally, culture can play a role as leisure physical activity may not be routine in some cultures or countries. Infrastructure and availability of safe and pleasant places to exercise, such as parks and green spaces, particularly in urban regions can encourage leisure physical activity but require considerable planning and come at a significant cost, highlighting the importance of urban planning in addressing these challenges [43]. Furthermore, differences between geographical regions and urban/rural areas may exist [7]. Individuals living in hills or mountainous areas may naturally engage in physical activity through activities such as walking up and down hills, although they might not always recognize these activities as formal exercise. Consequently, targeted interventions that address specific domains of physical activity are warranted, with a particular emphasis on prioritizing initiatives targeted toward promoting leisure‐time physical activity.

Comparisons with studies from other South Asian countries reveal similar patterns in the relationship between physical activity, obesity, and T2DM [44, 45, 46]. Rapid urbanization and urban migration are recognized as significant contributors to the increasing prevalence of low physical activity, overweight/obesity and T2DM in Nepal, where sedentary lifestyles, changes in dietary patterns, unplanned urbanization and housing, lack of infrastructure for physical activity, and traffic congestion collectively contribute to this obesogenic environment [7, 47, 48]. Furthermore, socioeconomic advancement leading to a shift from manual to sedentary jobs, and the availability of technological conveniences that reduce active transport methods have facilitated a rise in leisure time sedentary behavior [48, 49, 50, 51]. While these comparisons contribute to our understanding of the broader regional context of the NCD epidemic, the ongoing economic growth and urbanization in Nepal are expected to continue to impact physical activity patterns. Without effective public health initiatives to promote physical activity and reduce sedentary behavior, a continuing decline in overall physical activity levels is anticipated, potentially accelerating the prevalence of overweight/obesity, T2DM and other NCDs [24]. Therefore, adopting a multisectoral approach is crucial for devising comprehensive strategies to increase physical activity and reduce the incidence of overweight, obesity, and T2DM [44, 52].

Community‐based physical‐activity programs such as “Walk to Work” initiatives or creating more public green space can encourage regular exercise [53]. Initiatives focused on increasing awareness, accessibility, and affordability of physical activity opportunities are also important to stem the tide of overweight/obesity and T2DM [34, 40]. Cultural and environmental factors play a pivotal role in shaping physical activity behaviors in Nepal [22, 54]. Understanding these influences is vital for designing culturally sensitive and contextually relevant interventions. Tailored interventions should address the unique sociodemographic and cultural contexts of different regions within Nepal, considering the diverse lifestyle factors influencing physical activity patterns [22]. Moreover, interventions should not only focus on promoting physical activity but also consider the intricate interplay between lifestyle, environmental, and cultural contexts that shape behaviors in Nepal [54]. A holistic approach that integrates these multifaceted factors into intervention strategies is crucial for achieving sustainable and impactful outcomes in the promotion of physical activity and the management of overweight/obesity and T2DM [34, 40].

There are certain limitations to this study, including its cross‐sectional design, which limits our ability to establish causality between physical activity, obesity and T2DM. Future longitudinal studies are needed to assess the efficacy of physical activity promotion programs and interventions in reducing the risk of obesity and T2DM. The reliance on self‐reported data, particularly for physical activity, may introduce risks of recall and social desirability biases, potentially leading to under or overestimation of actual physical activity levels. Work‐related physical activity was assessed only in terms of paid and unpaid employment and may not fully capture other sources of physical activity, such as household chores. Similarly, leisure physical activity had low variability in the data, with the majority of participants reporting no activity in this domain. Despite these limitations, this study has several notable strengths. The large and diverse sample size enhances the reliability and validity of the findings, providing a comprehensive overview of the population of the nation as a whole. The use of a multistage cluster sampling technique ensures a representative sample, leading to more accurate and generalizable conclusions. Moreover, this study contributes valuable insights into NCD trends and risk factors in a developing country context, thereby contributing to the global understanding of health issues.

## 5 Conclusion

This study provides evidence on the association between physical activity, overweight/obesity and T2DM among adults in Nepal, based on a comprehensive analysis of a nationally representative NCD Risk‐Factor Surveillance data. The findings reveal an overall very low level of leisure time physical activity as well as a high prevalence of overweight/obesity and T2DM among Nepalese adults, with low physical activity levels significantly associated with these conditions. The low levels of physical activity and high levels of sedentary time are concerning, especially as they seem to be associated with higher levels of overweight/obesity and T2DM. This underscores the urgent need for public health interventions aimed at promoting regular physical activity and reducing sedentary behavior to help tackle the growing burden of overweight/obesity, T2DM and NCDs and improve health outcomes across the nation.

## Author Contributions

R.C., K.R. and M.K.P. contributed to the study concept and design. R.C. and K.R. curated data, conducted formal analysis, and provided project administration, resources, and supervision. J.A. and A.B. assisted with analysis and writing the original draft. S.J.A., P.P., L.R. and M.K.P. were involved in validation, critical review and editing manuscript. All authors had final approval of the submitted version.

## Conflicts of Interest

The authors declare no conflicts of interest.

## Data Availability

The data that support the findings of this study are available from World Health Organization. Restrictions apply to the availability of these data, which were used under license for this study. Data are available from https://ghdx.healthdata.org/record/nepal‐steps‐noncommunicable‐disease‐risk‐factors‐survey‐2019 with the permission of World Health Organization.
